# B-Cells and Plasmablasts as Architects of Autoimmune Disease: From Molecular Footprints to Precision Therapeutics

**DOI:** 10.3390/cells15020119

**Published:** 2026-01-09

**Authors:** Julie Sarrand, Muhammad Soyfoo

**Affiliations:** Department of Rheumatology, Hôpital Universitaire de Bruxelles (HUB), Université Libre de Bruxelles (ULB), 1070 Brussels, Belgium; julie.sarrand@ulb.be

**Keywords:** B cells, plasmablasts, autoimmune disease, B-cell endotypes, extrafollicular pathway, germinal center, autoantibodies, precision medicine, CAR-T therapy, immunomonitoring

## Abstract

**Highlights:**

**What are the main findings?**

- B-cell populations in systemic autoimmune diseases can be classified into distinct immunological endotypes—extrafollicular/IFN-high, germinal center/plasma cell-anchored, BAFF-dependent, and tissue-conditioned/fibro-inflammatory—each characterized by specific biomarker signatures, autoantibody profiles, and biological vulnerabilities.
- IgG subclass distribution (IgG1/IgG3 versus IgG4 predominance) critically determines therapeutic response patterns, with IgG4-mediated diseases showing rapid responses to B-cell depletion due to short-lived plasmablast dependence, while IgG1/IgG3-dominant diseases often resist anti-CD20 therapy due to long-lived plasma cell persistence.

**What are the implications of the main findings?**

- Endotype-based patient stratification enables mechanism-aligned therapeutic selection—directing JAK inhibitors and anti-CD19 therapies toward extrafollicular-dominant patients, proteasome inhibitors or CAR-T towards plasma cell-anchored disease, and BAFF inhibitors toward BAFF-dependent phenotypes—potentially improving response rates and reducing treatment failures.
- The convergence of B-cell endotyping frameworks across organ-specific and systemic autoimmune diseases suggests that shared immunological architectures, rather than traditional diagnostic boundaries, may better guide precision medicine approaches in clinical practice.

**Abstract:**

B-cells and plasmablasts have emerged as central organizers of autoimmune pathogenesis, extending far beyond their classical role as antibody-producing cells to orchestrate immune circuits, tissue microenvironments, and therapeutic trajectories. Advances in single-cell technologies, high-dimensional cytometry, and B-cell receptor sequencing have uncovered a dynamic continuum of B-cell differentiation programs that drive clinical heterogeneity across systemic autoimmune diseases. Plasmablasts, in particular, have gained recognition as highly responsive sensors of immune activation: they expand during flares, encode interferon-driven and extrafollicular responses, and correlate with disease severity. Autoantibody profiles, long viewed as static diagnostic signatures, are now understood as durable molecular footprints of distinct B-cell pathways. In this review, we propose an endotype-based framework integrating B-cell circuits with clinical phenotypes, illustrate therapeutic decision-making through mechanistic case vignettes, and outline future strategies combining immunomonitoring, multi-omics, and precision therapeutics. We further address translational challenges and discuss complementary approaches, including T-cell modulation, FcRn inhibition, and antigen-specific tolerization.

## 1. Introduction: The B-Cell Renaissance in Autoimmune Disease

For decades, B-cells were primarily viewed as terminal producers of autoantibodies in autoimmune disease. This narrow framing reduced B-cell biology to a downstream effector function and placed antibody titers at the center of disease classification and therapeutic monitoring. Yet clinical reality has persistently contradicted this simplification: patients with comparable autoantibody profiles experience divergent disease trajectories, organ involvement, and treatment responses, underscoring that humoral immunity alone cannot account for autoimmune heterogeneity [1,2,3].

This paradigm has been profoundly reshaped by the emergence of single-cell transcriptomics, high-dimensional cytometry, and B-cell receptor sequencing. These approaches have revealed B-cells as dynamic immune hubs that integrate signals from interferons, cytokines, T-cell interactions, and stromal niches. Beyond antibody secretion, B-cells regulate cytokine networks, organize ectopic lymphoid structures, present antigen to autoreactive T-cells, and imprint tissue-selective inflammatory programs [3,4,5]. Rather than following a linear differentiation hierarchy, B-cells populate a plastic continuum of functional states shaped by antigen affinity, metabolic programming, local microenvironment, and inflammatory context [6,7].

A central insight emerging from this revised framework is the existence of distinct B-cell circuits across autoimmune diseases. Interferon-amplified extrafollicular responses, germinal center-dependent affinity maturation, BAFF-driven naïve compartment expansion, and stromal-conditioned fibrotic programs represent biologically coherent pathways that govern plasmablast dynamics, autoantibody architectures, and therapeutic sensitivity [8,9,10,11,12]. These circuits provide a mechanistic explanation for why B-cell-targeted therapies yield heterogeneous outcomes across seemingly similar clinical phenotypes.

Equally transformative has been the recognition that B-cell activity is deeply shaped by tissue microenvironments. Stromal cells, fibroblasts, and specialized endothelial networks orchestrate B-cell survival, localization, and differentiation, while ectopic lymphoid structures serve as autonomous immune niches in chronically inflamed tissues. These tissue-embedded programs are now recognized as major drivers of disease persistence and therapeutic resistance in systemic autoimmunity [4,13,14,15].

In this review, we propose an endotype-based model positioning B-cells and plasmablasts not as passive effectors but as architects of autoimmune disease. We integrate cellular immunology with clinical reasoning to link molecular programs to patient-level phenotypes and therapeutic logic. By interpreting autoantibodies as molecular footprints of B-cell fate decisions and plasmablasts as high-resolution indicators of immune-circuit activity, we aim to establish a conceptual framework for precision medicine in systemic autoimmunity. Finally, we address translational challenges and emerging strategies to operationalize B-cell endotyping in routine clinical practice.

This review first revisits B-cell biology using high-dimensional and single-cell frameworks, then integrates human tissue and immune repertoire data to define reproducible B-cell endotypes in systemic autoimmunity. We subsequently discuss plasmablasts as dynamic biomarkers and autoantibodies as molecular footprints of underlying immune circuits. Finally, we map these endotypes to therapeutic vulnerabilities, address translational challenges, and extend the framework to comparisons with organ-specific autoimmune diseases and future precision strategies.

## 2. B-Cell Biology Revisited: From Subsets to High-Dimensional Ecosystems

Classical immunology classified B-cells into discrete developmental stages, naïve, memory, germinal center (GC), plasmablast, and plasma cell, based on surface markers and presumed functional stability. While useful, this taxonomy fails to capture the intrinsic adaptability of the B-cell lineage. Converging evidence now indicates that B-cells populate dynamic transcriptional and functional landscapes rather than fixed states, both in physiological immunity and autoimmune pathology [6,7].

Single-cell transcriptomics, high-dimensional cytometry, and spatial profiling have revealed an unexpected degree of heterogeneity within canonical B-cell compartments. Human single-cell atlases in systemic autoimmune diseases demonstrate disease-specific transcriptional states of B-cells, including interferon-enriched and extrafollicular signatures in affected tissues [16,17,18]. Memory B-cells comprise multiple states with distinct effector programs, survival properties, and tissue tropisms, while plasmablasts span a continuum of metabolic and functional phenotypes that track with inflammatory activity [17,18,19,20]. B-cell identity therefore emerges from the integration of molecular circuits, tissue context, and inflammatory tone rather than from static phenotypic categories [6,7].

### 2.1. Determinants of B-Cell Fate

B-cell differentiation is not linear but governed by interacting regulatory axes that jointly specify cell identity.

At the transcriptional level, a tightly coordinated network involving BCL6, BLIMP-1, IRF4, and T-bet orchestrates commitment toward GC maturation, plasma-cell differentiation, or extrafollicular inflammatory programs [8,9,10,21]. Cytokine signaling establishes a second layer of control. BAFF supports naïve and transitional survival, IL-21 sustains GC reactions, type I and II interferons promote extrafollicular activation, and TGF-β contributes to tissue-imprinted phenotypes characteristic of chronic inflammation [7,22,23].

Cellular metabolism represents a decisive regulatory node. Plasmablast differentiation is accompanied by a metabolic switch toward glycolysis to fuel rapid immunoglobulin synthesis, whereas GC B-cells rely on oxidative phosphorylation and lipid metabolism during affinity maturation [24,25].

B-cell receptor (BCR) signaling further constrains lineage trajectories. Antigen affinity, persistence, and receptor engagement dynamics distinguish GC-dependent maturation from extrafollicular responses. Large-scale repertoire analyses reveal recurrent structural patterns among autoreactive clones, supporting the existence of convergent pathogenic programs in autoimmunity [1,2,7].

Finally, tissue context imposes a spatial dimension to B-cell fate. Stromal cells, chemokine gradients, and extracellular matrix components define survival niches and migration patterns, introducing organ-specific immune architectures that can remodel B-cell behavior independent of the systemic compartment [5,13,15,19].

### 2.2. Germinal Center and Extrafollicular Circuits

A fundamental organizing principle of humoral immunity is the dichotomy between GC-dependent and extrafollicular (EF) pathways.

Germinal centers provide spatially organized platforms for somatic hypermutation and affinity-based selection, generating high-affinity memory B-cells and long-lived plasma cells that sustain antibody production for decades [9,26,27]. Plasma cells ultimately integrate into specialized bone-marrow niches, conferring durability to humoral immunity and, in autoimmune disease, to pathogenic autoantibody production [27,28].

In contrast, EF responses represent a rapid, innate-coupled axis of B-cell activation. Triggered by toll-like receptors, interferons, and inflammatory cytokines, this pathway bypasses conventional tolerance checkpoints and generates bursts of short-lived plasmablasts with limited somatic mutation [7,8,12].

These circuits are not mutually exclusive. Instead, they coexist within hybrid immune states that plausibly explain the incomplete and transient efficacy of current B-cell-directed therapies across autoimmune diseases [6,7,12].

### 2.3. T-Bet^+^ and Double-Negative B-Cells as Pathogenic Intermediates

Age-associated and double-negative B-cell populations represent a cellular manifestation of EF bias. These cells express T-bet, respond robustly to interferon and TLR7 signaling, and exhibit increased autoreactivity [8,20].

In systemic lupus erythematosus, expansion of this compartment correlates with plasmablast output, tissue infiltration, and disease activity [18,20].

At sites of organ damage, such as the kidney, chemokine gradients generated by tissue macrophages actively recruit CXCR3-positive plasmablasts, promoting their differentiation into long-lived autoantibody-producing cells [17].

Human genetics provides direct causality. Gain-of-function variation in TLR7 results in monogenic lupus syndromes characterized by massive expansion of T-bet-positive B-cells and enforced extrafollicular differentiation, establishing this program as a primary driver of autoimmunity [21].

### 2.4. From Cellular Taxonomy to Systems Immunology

Collectively, these findings compel a shift from static taxonomy toward a systems view of B-cell biology. B-cells operate as adaptive networks shaped by transcriptional architecture, metabolic state, and tissue context rather than phenotype alone [6,7,15].

This framework fundamentally alters how B-cell-targeted therapies should be interpreted. Agents directed at CD20, BAFF, interferon signaling, FcRn-mediated IgG recycling, or plasma-cell survival modulate distinct nodes of the B-cell ecosystem, and their clinical efficacy depends critically on which circuit dominates in a given patient [7,12,29].

A systems-level understanding of B-cell biology is therefore not optional—it is the prerequisite for precision immunotherapy.

## 3. B-Cell Ecosystems in Human Autoimmunity

**Figure 1:**
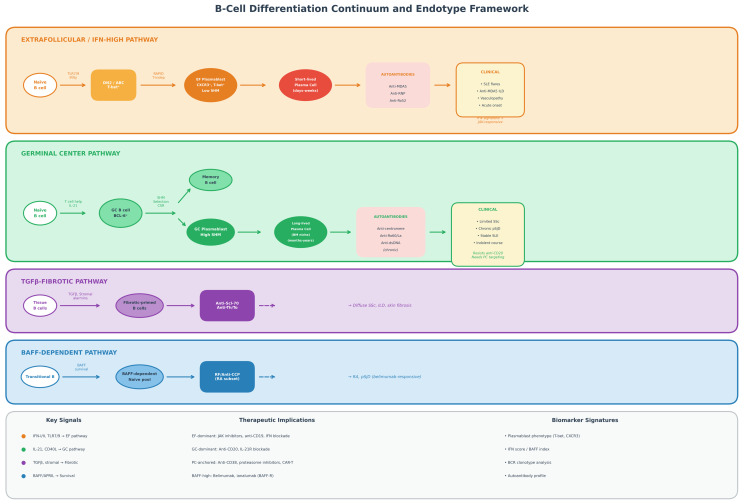
B-Cell Differentiation Continuum and Endotype Framework. Schematic representation of dominant immunoregulatory patterns governing B-cell differentiation in systemic autoimmune diseases. Four major B-cell programs are illustrated as overlapping and context-dependent circuits rather than rigid lineages. The interferon-amplified extrafollicular circuit (red) is characterized by expansion of DN2/ABC populations and short-lived plasmablast differentiation under strong innate immune pressure. The germinal center-associated circuit (green) supports affinity maturation and generation of class-switched memory B-cells and long-lived plasma cells. The stromal-conditioned fibro-inflammatory module (orange) reflects tissue-embedded B-cell states shaped by local microenvironmental signals including TGF-β. The BAFF-skewed survival axis (purple) highlights conditions in which B-cell homeostasis is driven by BAFF-dependent selection and persistence of naïve and transitional cells. These circuits are associated with distinct but overlapping autoantibody patterns, clinical features, and therapeutic vulnerabilities, illustrating how molecular programs rather than diagnostic labels may define mechanistic endotypes. This figure represents a conceptual synthesis based on the available literature rather than a deterministic classification. Abbreviations: ABC, Age-Associated B-Cell; BAFF, B-Cell Activating Factor; DN2, Double-Negative 2 B-Cell; EF, Extrafollicular; GC, Germinal Center; IFN, Interferon; pSjD, Primary Sjögren’s Disease; SLE, Systemic Lupus Erythematosus; SSc, Systemic Sclerosis; TGF-β, Transforming Growth Factor Beta.

The conceptual distinction between germinal center and extrafollicular circuits provides a useful framework (Figure 1), but recent human datasets demonstrate that B-cell biology in autoimmunity is best understood as tissue-embedded ecosystems rather than circulating subsets. Single-cell RNA sequencing, spatial transcriptomics, and immune repertoire profiling reveal disease-specific B-cell architectures that cannot be inferred from blood alone [16,18,30,31].

Across systemic autoimmune diseases, these approaches identify recurring patterns including interferon-driven extrafollicular programs, structured tertiary lymphoid niches, and clonal networks bridging circulation and tissue [18,32,33].

### 3.1. Human Single-Cell Mapping of B-Cell States

In lupus nephritis, single-cell transcriptomics has demonstrated a dominant extrafollicular B-cell and plasmablast program directly within the kidney, associated with granzyme K-positive CD8 T-cells and local chemokine cues [18]. These tissue-resident B-cell states exhibit transcriptional activity distinct from circulating populations, including enhanced antigen presentation and survival modules.

In primary Sjögren disease, multimodal single-cell profiling has revealed parallel depletion of memory B-cells in blood and accumulation of activated B-cells in salivary tissue [16]. Single-cell atlases of labial salivary glands further demonstrate that B-cell clusters expressing interferon and plasma-cell programs are anatomically colocated with follicular helper T-cells and activated fibroblasts [30,31].

Recent spatial profiling of anti-SSA-associated disease demonstrates that autoantibody specificities are associated with distinct BCR clonotypes and reproducible micro-anatomical positions within glandular lesions, linking antibody profiles to tissue-level immune architecture [32].

Collectively, these studies position autoimmune diseases as organ-specific B-cell ecosystems rather than disorders of generic circulating subsets.

### 3.2. Tissue Niches and Tertiary Lymphoid Structures

B-cells in autoimmune tissues organize within tertiary lymphoid structures (TLS) that recapitulate features of secondary lymphoid organs. Classic studies first framed TLS as ectopic immune organs able to support affinity maturation and class switching [33].

Recent data reframe TLS as stromal-driven structures, in which fibroblasts, endothelial networks and perivascular cells produce CXCL13, CCL19 and survival factors that assemble lymphoid architecture [5,13]. Reviews across organs demonstrate that TLS formation is consistently associated with disease chronicity and therapeutic resistance in autoimmunity, in sharp contrast to its often favourable prognostic significance in cancer [34,35].

In lupus nephritis, renal TLS correlate with chronicity indices and provide niches for autoreactive B-cells and plasmablast survival [36].

These findings establish TLS as architectural engines of persistent autoimmunity.

### 3.3. Clonal Architecture and Immune Repertoire

High-throughput BCR sequencing demonstrates that autoimmunity is associated with stereotyped repertoire features including biased V-gene usage, elongated CDR3 regions and restricted clonal diversity [37]. Repertoire analysis shows that tolerance failure occurs at multiple checkpoints, including early B-cell development, germinal center selection and plasmablast differentiation [38].

Integration of BCR data with single-cell transcriptomics demonstrates that distinct B-cell subsets carry characteristic clonal imprints that correlate with disease phenotype and therapeutic response [39]. CD21-low B-cells represent a convergence of phenotype and clonal risk, with enrichment for autoreactive and antigen-experienced clones [40].

Machine learning approaches applied to immune receptor repertoires now permit disease classification based purely on BCR and TCR architecture, demonstrating that autoimmunity leaves a detectable molecular footprint in clonal structure [41].

### 3.4. Extrafollicular Circuits as Shared Autoimmune Modules

Experimental models demonstrate that extrafollicular responses alone are sufficient to initiate lupus-like disease in the absence of functional germinal centers [42].

In human disease, this circuit extends beyond lupus. In childhood idiopathic nephrotic syndrome, extrafollicular B-cell activation dominates during relapse and is sensitive to B-cell depletion [43].

Mechanistically, CD21 signaling primes autoreactive B-cells toward extrafollicular differentiation, establishing this pathway as an active decision process rather than a default failure mode [44]. A recent conceptual framework further formalizes extrafollicular responses as defined developmental pathways with specific phenotypic and functional criteria [12].

### 3.5. From Ecosystems to Endotypes

Integration of single-cell profiling, spatial transcriptomics and immune repertoire analysis suggests that B-cell biology partitions into reproducible endotypes rather than clinical categories.

Three dominant patterns emerge across diseases:

An interferon-driven extrafollicular endotype characterized by DN2 and CD21-low B-cells with high plasmablast output is prominent in lupus nephritis and systemic flares [12,18,42].

A stromal-driven TLS endotype with germinal center-like structures dominates in Sjögren disease, rheumatoid arthritis and fibrotic phenotypes [5,13,16,33,34,35,36].

A plasma-cell persistent endotype characterized by long-lived autoantibody production with limited ongoing B-cell activation defines late-stage and treatment-refractory disease [36].

Recent work integrating blood immunophenotyping, renal single-cell data and proteomics confirms that peripheral B-cell states reflect tissue-level immune architecture, establishing endotyping as clinically tractable [45].

## 4. Plasmablasts as Dynamic Biomarkers of Immune Activation

**Figure 2:**
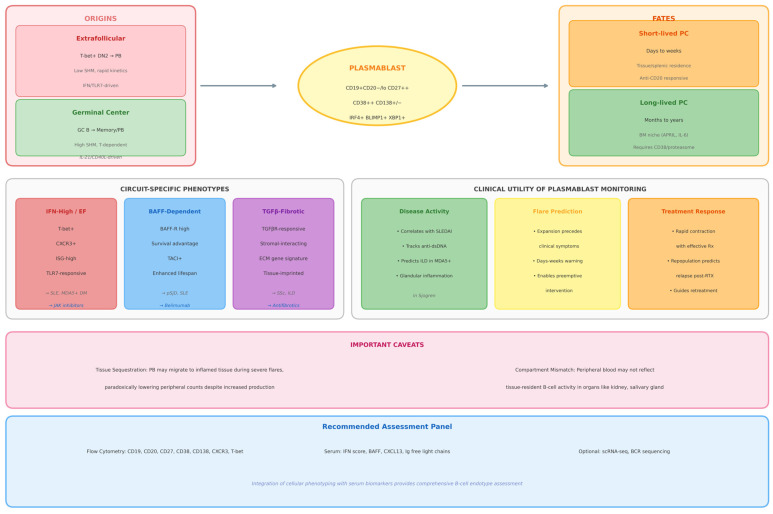
Plasmablast Biology and Clinical Biomarker Utility. Schematic overview of plasmablast origins, phenotypes and clinical relevance in systemic autoimmune diseases. The central panel depicts the plasmablast immunophenotype (CD19^+^CD20^−^/loCD27^++^CD38^++^CD138^+^/^−^). The origins panel contrasts extrafollicular differentiation under interferon and TLR-dominated conditions with germinal center-dependent maturation. The fates panel illustrates short-lived versus long-lived plasma cell trajectories. Circuit-associated phenotypes highlight how plasmablast features reflect dominant immune programs rather than diagnostic categories. Clinical utility panels summarize how plasmablast monitoring may inform disease activity, flare prediction and therapeutic response. This figure represents a conceptual synthesis rather than a deterministic model. Abbreviations: PB, Plasmablast; PC, Plasma Cell; EF, Extrafollicular; GC, Germinal Center; IFN, Interferon; BAFF, B-cell Activating Factor; TGF-β, Transforming Growth Factor Beta; SLE, Systemic Lupus Erythematosus; pSjD, Primary Sjögren’s Disease; SSc, Systemic Sclerosis; DM, Dermatomyositis.

Plasmablasts have moved from being viewed as short-lived effector cells to being recognized as high-value, circuit-specific biomarkers in systemic autoimmunity. Their clinical interest rests on three core properties (Figure 2). First, plasmablasts display rapid response kinetics, expanding within days in response to flares or therapeutic interventions. Second, their phenotype reflects the dominant inflammatory circuit, for example, interferon-driven extrafollicular versus BAFF-dependent or germinal center-derived responses. Third, their dynamics correlate with disease activity and treatment outcomes across several autoimmune diseases [46,47].

In SLE, multiple cohorts now show that peripheral plasmablast or CD27^high^CD38^high^ plasma cell frequencies correlate with SLEDAI scores, anti-dsDNA titers, complement consumption and classical acute phase reactants, and that plasmablast proportions provide better discrimination of active disease than traditional serologic markers alone [46,47,48]. Longitudinal monitoring further suggests that sustained plasmablast elevation after therapy is associated with refractory disease, whereas early declines track with clinical remission and steroid-sparing responses [46,47].

Beyond simple enumeration, plasmablast phenotypes report on the upstream pathway that generated them. Interferon- and TLR7-driven extrafollicular circuits preferentially generate T-bet^+^, CXCR3^+^, CD11c^+^ antibody-secreting cells enriched in interferon-stimulated gene signatures and related age-associated or DN2-like B-cell intermediates [36,45,49]. In contrast, chronic BAFF exposure and BAFF-blocking therapy reshape naïve and transitional compartments, selectively contracting autoreactive plasmablast outputs and altering survival signals through BAFF-R and APRIL pathways, which is reflected in changing plasmablast frequencies under belimumab or dual BAFF/APRIL blockade [42,50]. Metabolic and transcriptional profiling further distinguishes short-lived inflammatory plasmablasts from cells transitioning toward long-lived plasma cell fates, with unfolded protein response and proteasome-dependency signatures highlighting subsets that may be particularly susceptible to proteasome inhibition or CAR-T-mediated depletion [43,45].

A key caveat in interpreting circulating plasmablast counts is tissue sequestration. Chemokine axes such as CXCR3–CXCL9/10 and CXCR4–CXCL12 direct plasmablast homing to inflamed organs and survival niches in bone marrow and chronically inflamed tissues. In models of lupus nephritis, Cxcl9^high^ macrophages recruit Cxcr3^+^ plasmablasts into the kidney, and CXCL9 neutralization redistributes these cells from renal tissue back to the circulation while improving renal pathology [19]. Similar principles likely apply to other tissue-targeted autoimmune diseases, where low peripheral plasmablast counts may paradoxically reflect intense tissue recruitment rather than quiescent disease. Integrating plasmablast dynamics with tissue-level data, chemokine signatures and conventional biomarkers is therefore essential before using plasmablasts as stand-alone readouts of disease activity.

## 5. Autoantibodies as Molecular Footprints of B-Cell Programs

Autoantibodies have long underpinned diagnosis and classification in systemic autoimmune diseases, but their biological meaning goes beyond static disease labels (Table 1). Contemporary data support a view of autoantibodies as durable molecular footprints of the B-cell programs that produced them, integrating antigen availability, cytokine milieu, BCR selection pressure and tissue context rather than reflecting a single linear pathway [7,20,51].

Autoantibodies such as anti-MDA5, anti-U1RNP and anti-Ro52/TRIM21 are frequently enriched in interferon-dominant, flare-prone disease states and often coexist with expanded plasmablast and DN2/ABC-like B-cell compartments. In SLE and related interferon-driven conditions, repertoire and phenotypic analyses demonstrate that a fraction of autoreactive specificities arises from extrafollicular responses, characterized by limited somatic hypermutation, broad clonal expansion and plasmablast-biased kinetics, consistent with a rapid, innate-coupled origin [20,51,52]. These profiles are typically associated with abrupt disease flares, vasculopathic or lung-dominant phenotypes and high interferon gene signatures, although substantial inter-individual heterogeneity remains [18,20].

By contrast, classical specificities such as anti-centromere, anti-La (SS-B), anti-Ro60 (SS-A) and chronic-phase anti-dsDNA antibodies are more consistently associated with germinal center-driven maturation and persistence through long-lived plasma cells. Germinal center biology provides a mechanistic explanation for their high levels of somatic hypermutation, stable titers over years and resistance to CD20-directed therapies, since long-lived plasma cells lack CD20 expression and reside in protective bone marrow or tissue niches [7,27,53].

In fibrosing and tissue-remodeling syndromes, including systemic sclerosis, interstitial lung disease and chronic salivary-gland involvement, autoantibody patterns (for example, anti-Scl-70, anti-PM/Scl, anti-Th/To) often arise in microenvironments where fibroblasts, endothelial cells and stromal networks orchestrate chronic inflammation. Studies of tertiary lymphoid structures in fibrotic tissues show that B-cells co-localize with activated stroma and are exposed to mediators such as TGF-β, CXCL13 and survival factors that can shape their activation and fate [4,5,33]. While a discrete “TGF-β B-cell pathway” has not been formally defined, these data support a model in which tissue-conditioned B-cell states contribute to fibrotic endotypes and associated autoantibody profiles rather than a purely systemic, circulation-defined process [4,5,33].

Taken together, autoantibodies are best interpreted as probabilistic signatures of underlying B-cell circuits rather than deterministic barcodes of a single pathway. Their full informational content emerges when they are integrated with immunophenotyping, single-cell and spatial transcriptomics, and BCR repertoire analysis. In this frame, autoantibodies become key components of an endotype-based approach, helping to anchor clinical phenotypes to specific B-cell programs and to guide mechanism-informed therapeutic strategies [7,51,52,53].

## 6. Translational Scenarios: How B-Cell Endotypes Shape Disease Architecture

**Figure 3:**
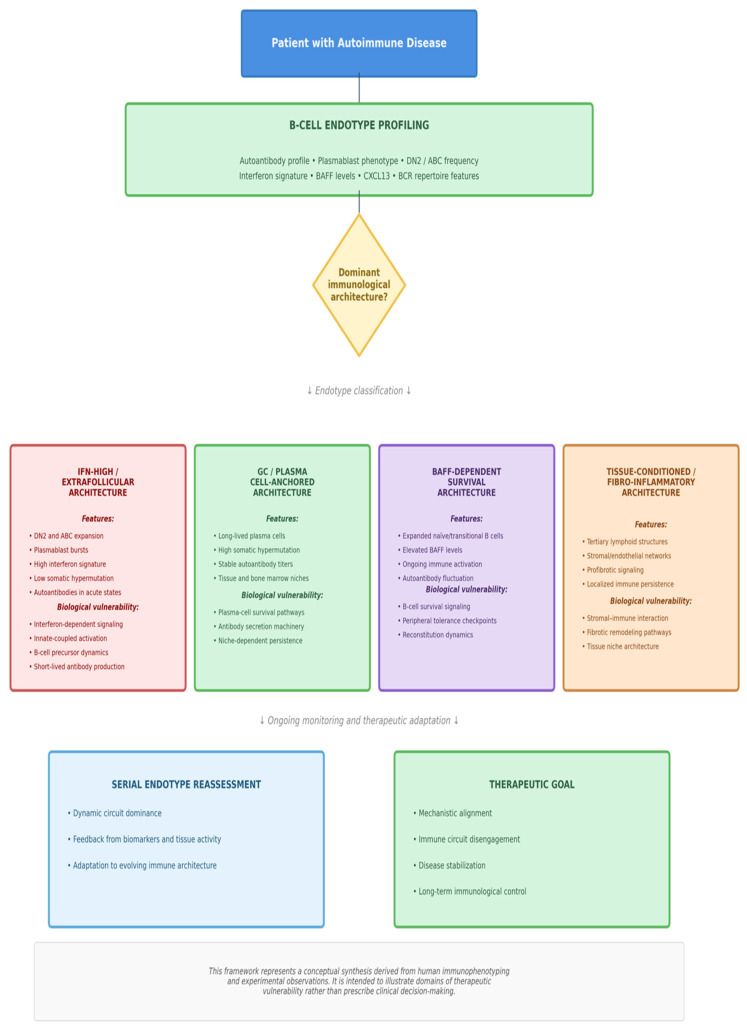
Endotype-Based Framework for Therapeutic Reasoning in B-Cell-Mediated Autoimmunity. Conceptual model illustrating how dominant B-cell endotypes define distinct immunological architectures associated with specific biological vulnerabilities. Following integrated profiling of circulating and tissue-level biomarkers including autoantibody signatures, plasmablast phenotypes, interferon scores, BAFF levels, and B-cell receptor repertoire features, four major immune programs emerge: interferon-driven extrafollicular activation, germinal center and plasma cell-anchored persistence, BAFF-dependent survival bias, and tissue-conditioned fibro-inflammatory ecosystems. These architectures represent domains of mechanistic vulnerability rather than prescriptive treatment algorithms. The framework emphasizes dynamic reassessment and circuit dominance rather than static diagnostic classification. Abbreviations: DN2, double-negative 2 B-cell; GC, germinal center; IFN, interferon.

To illustrate how B-cell endotypes can inform mechanistic interpretation of clinical phenotypes, we outline two paradigm scenarios derived from human immunophenotyping, single-cell analyses and tissue-based studies. These scenarios are not treatment recommendations; they are conceptual frameworks linking immune architecture to therapeutic vulnerabilities (Figure 3).

### 6.1. Scenario A: Interferon High, Extrafollicular Dominant Architecture

This scenario corresponds to patients with abrupt inflammatory flares, strong type I or type II interferon signatures and prominent plasmablast expansion in blood. Across systemic autoimmune diseases, interferon-driven states are associated with expansion of DN2 or ABC-like B-cell populations, oligoclonal BCR repertoires with limited somatic hypermutation and transcriptional programs consistent with extrafollicular activation rather than germinal center selection [7,20,27,53].

Mechanistically, B-cells integrate interferon signals, TLR7 and TLR9 engagement and inflammatory cytokines to bypass germinal centers and differentiate rapidly into short-lived plasmablasts. In this architecture, disease activity is driven more by accelerated innate coupled circuits than by long-term affinity-matured memory. Autoantibodies are produced in bursts by plasmablasts rather than by stable pools of long-lived plasma cells [20,27,53].

From a conceptual standpoint, this endotype is predicted to be preferentially sensitive to interventions that interrupt interferon-dependent signalling and deplete activated precursors in the DN2 and naive or memory compartments, whereas strategies targeting long-lived plasma cells alone are less likely to modify short-term disease activity in the absence of broader circuit-level modulation [7,20,53].

### 6.2. Scenario B: Tissue Anchored, Stromal Conditioned and Plasma Cell Supported Architecture

This scenario reflects chronic autoimmune states in which autoantibody titers remain remarkably stable over time while peripheral B-cell activation appears modest. Single-cell and spatial analyses across organs show that in such settings, B-cells accumulate within tertiary lymphoid structures embedded in fibroblast-rich stroma, where stromal, endothelial and myeloid cells provide chemokines, survival factors and profibrotic mediators that organize long-term immune niches [4,5].

Within these tissue ecosystems, germinal center-like reactions and plasma cell differentiation occur in situ, and long-lived plasma cells integrate into bone marrow or tissue niches that are largely insulated from fluctuations in circulating B-cells. The resulting serological stability and therapeutic resistance of established autoantibody profiles are better explained by plasma cell biology and stromal support than by ongoing systemic B-cell activation [4,5,7,33].

Conceptually, this endotype is less likely to respond to isolated peripheral B-cell depletion and more likely to require strategies that either disrupt plasma cell survival niches or modulate stromal immune crosstalk at the tissue level. In this framework, endotype-informed reasoning shifts the focus from diagnostic labels toward the underlying immune architecture that constrains clinical trajectories and shapes differential sensitivity to targeted interventions [4,5,7].

## 7. B-Cell-Directed Therapies as Endotype-Dependent Biological Interventions

The therapeutic landscape in systemic autoimmunity has expanded rapidly, yet clinical efficacy remains highly heterogeneous. A unifying principle emerging from human immunophenotyping is that therapeutic response depends primarily on alignment between the intervention and the dominant immune architecture rather than on drug class per se [2,54]. B-cell-directed agents therefore act not as universal suppressors but as circuit modulators whose impact is constrained by endotype (Table 2).

### 7.1. Anti-CD20 Monoclonal Antibodies

CD20-directed antibodies deplete mature B-cells but spare early precursors and antibody-secreting plasma cells. Their biological footprint is therefore restricted to endotypes in which memory B-cells and antigen presentation sustain pathogenic immunity [55,56]. In germinal center-driven disease, B-cell depletion disrupts immune reinforcement loops and alters T-cell crosstalk; in contrast, plasma-cell-anchored disease is largely resistant because long-lived plasma cells persist within protective niches [27,53,57]. Treatment failure in this context reflects immune architecture rather than insufficient depletion [57].

### 7.2. BAFF and BAFF-R Inhibition

BAFF-axis blockade selectively reshapes transitional and naïve B-cell compartments and alters post-depletion repopulation dynamics [58,59]. This explains preferential activity in BAFF-high endotypes with expanded immature pools and fluctuating serologies [60,61]. Rather than directly suppressing antibody secretion, BAFF inhibition tightens tolerance thresholds and constrains permissive survival landscapes [58,59,60,61].

### 7.3. Interference with Interferon-Dependent Circuitry

JAK inhibition does not primarily target B-cells but disrupts upstream signal integration nodes controlling inflammatory transcriptional programs [62]. Interferon-amplified extrafollicular endotypes, characterized by DN2 expansion and plasmablast bursts, show biological sensitivity to pathway-level signal disruption rather than lineage-restricted depletion [20,63,64]. Therefore, B-cell pathology may be attenuated indirectly by reshaping the inflammatory field that licenses aberrant fate decisions [20,62].

### 7.4. Plasma-Cell-Directed Vulnerabilities

Plasma-cell-targeted strategies address the cellular source of persistent serological disease through disruption of secretory programs and survival pathways [65,66,67]. However, stromal protection and niche architecture often limit durability, highlighting that immune reset is biologically constrained when tissue ecosystems remain intact [5,66,67].

### 7.5. Immune Reset with CAR-T Therapy

CD19-directed CAR-T therapy represents a maximal perturbation of immune architecture. Early human experience demonstrates that the collapse of autoreactive networks can permit immune tolerance reconstitution in selected patients, supporting the concept that a subset of autoimmune disease is fundamentally B-cell-driven [68,69,70,71]. Whether remission reflects loss of pathogenic memory, niche disruption, or immune re-education remains under investigation [70,71].

### 7.6. From Treatment Classes to Biologically Defined Vulnerability

Collectively, therapies map onto layers of immune organization: lineage depletion, survival checkpoint modulation, inflammatory-field control, plasma-cell dependency, and tissue architecture [2,5,54,61,66]. Framing interventions as exploitation of biological vulnerability rather than disease labels enables rational, mechanism-based strategies aligned with immune configuration.

### 7.7. IgG Subclass-Dependent Therapeutic Outcomes

The four IgG subclasses (IgG1, IgG2, IgG3, IgG4) differ fundamentally in their effector functions, with important implications for autoantibody pathogenicity and therapeutic response [72,73]. IgG1 and IgG3 demonstrate the highest affinity for Fcγ receptors and potently activate complement, antibody-dependent cellular cytotoxicity (ADCC), and phagocytosis, making them the predominant pathogenic subclasses in most systemic autoimmune diseases. In contrast, IgG4 exhibits weak FcγR binding and lacks complement-activating capacity, instead mediating pathogenicity through direct target blockade rather than inflammatory effector mechanisms [73]. IgG2 preferentially responds to polysaccharide antigens and has intermediate effector function [72].

These structural differences translate into distinct therapeutic vulnerabilities. In IgG4-predominant diseases such as pemphigus, MuSK myasthenia gravis, and IgG4-related disease, B-cell depletion with rituximab produces rapid clinical improvement accompanied by preferential reduction in serum IgG4 levels, suggesting that IgG4-secreting cells derive from short-lived plasmablast populations continuously replenished by circulating B-cells [74,75]. The swift therapeutic response in these conditions contrasts with IgG1/IgG3-dominant diseases, where long-lived plasma cells in protective niches sustain autoantibody production despite effective B-cell depletion. FcRn inhibitors accelerate catabolism of all IgG subclasses but may show differential efficacy depending on the half-life characteristics of each subclass, with IgG3 exhibiting the shortest natural half-life due to reduced FcRn binding affinity [72].

In systemic autoimmune diseases, serum IgG subclass profiling reveals disease-specific patterns. Primary Sjögren disease, SLE, and systemic sclerosis demonstrate elevated IgG1 and IgG3 relative to healthy controls, while IgG4 levels remain relatively unchanged, distinguishing these conditions from IgG4-related disease [76]. Within individual diseases, subclass distribution may further stratify patients: higher IgG1/IgG3 ratios correlate with complement-mediated tissue damage and may predict responsiveness to complement-targeted therapies, whereas IgG4 predominance in specific autoantibody populations (such as anti-desmoglein in pemphigus) identifies patients likely to respond rapidly to B-cell depletion [75]. Incorporating IgG subclass assessment into endotype frameworks may therefore refine therapeutic prediction and enable more precise matching of intervention to underlying immunopathology.

## 8. Beyond B-Cell Depletion: Complementary Immunomodulatory Strategies

The conceptual framework of B-cell endotypes naturally extends beyond lineage depletion toward strategies targeting the immune system at orthogonal levels, including T-cell help, antibody persistence, and immune tolerance. These approaches offer additional layers of intervention by reshaping immune architecture rather than directly removing B-cells.

### 8.1. Modulation of T-Cell Help and Immune Regulation

T-cell and B-cell biology are tightly coupled. Germinal center reactions critically depend on follicular helper T-cells, while regulatory T-cells (Tregs) constrain autoreactive B-cell activation. Therapeutic strategies that interfere with costimulatory pathways or expand regulatory compartments can therefore reshape humoral immunity through indirect mechanisms [77,78]. Costimulatory blockade modifies T–B synapse formation and alters assistance signals required for germinal center maintenance, while low-dose IL-2 preferentially expands Tregs and shifts tolerance thresholds. These approaches support the view that immune reconstitution following B-cell depletion is an active biological process that can be shaped by restoring dominant regulatory networks rather than by prolonged immunosuppression alone [79].

### 8.2. FcRn-Mediated IgG Clearance: Targeting Autoantibody Persistence

The neonatal Fc receptor (FcRn) regulates IgG homeostasis by rescuing IgG from lysosomal degradation and extending its half-life. Pharmacological inhibition of FcRn accelerates IgG catabolism, leading to a rapid reduction in circulating autoantibody levels without directly targeting B-cells [80,81]. This mechanism uncouples serological disease activity from upstream immune architecture and illustrates that autoantibody-mediated pathology can be biologically dissociated from immune cell persistence. FcRn inhibition therefore provides a complementary strategy that addresses effector burden while immune-directed therapies act on disease drivers [80].

### 8.3. Antigen-Specific Tolerization: Reprogramming Immune Memory

Induction of antigen-specific tolerance represents the theoretical endpoint of autoimmune therapy: targeted immune deletion or silencing without global immunosuppression. Experimental approaches include nanoparticle-based antigen delivery systems, tolerogenic dendritic cells, peptide–MHC complexes, and CAR-engineered Tregs designed to suppress autoreactive immune responses in an antigen-restricted manner [82,83]. These platforms aim not to suppress immunity but to re-educate it, selectively extinguishing autoreactive clones while preserving protective immune function. For B-cell-mediated disease, tolerogenic strategies offer the opportunity to shape immune reconstitution following depletion by guiding repertoire renovation rather than permitting stochastic rebound [77,84].

## 9. Practical Challenges for Clinical Implementation of B-Cell Endotyping

### 9.1. Accessibility of Advanced Immunophenotyping Technologies

High-dimensional B-cell profiling methods, including multiparameter flow cytometry, immune-repertoire sequencing, and single-cell transcriptomics, remain largely confined to specialized research centers or tertiary referral hospitals. Although flow cytometry platforms are available in most academic institutions, advanced panels for B-cell phenotyping, as well as downstream computational expertise, are not universally accessible. Immune-repertoire sequencing and single-cell technologies require dedicated infrastructure, bioinformatics pipelines, and technical expertise that limit broad implementation beyond reference laboratories. While costs are progressively decreasing, scalability remains a major barrier to routine clinical deployment [85,86,87].

### 9.2. Standardization and Inter-Laboratory Reproducibility

Lack of standardization currently represents a critical obstacle. Variability in antibody panels, gating strategies, cytometer calibration, and reference ranges results in poor comparability across laboratories. Initiatives such as the EuroFlow consortium have demonstrated that highly standardized protocols and harmonized antibody panels can enable reproducible immunophenotyping across centers [88,89]. External quality assurance programs further contribute to reproducibility and data reliability [90], but such frameworks are not yet routinely implemented in autoimmune disease diagnostics. Without standardized workflows, B-cell-based endotyping cannot be reliably translated from research settings to clinical practice.

### 9.3. Integration of Multidimensional Data and Clinical Interpretation

B-cell endotype classification ideally integrates cellular phenotypes, immune repertoire features, serum biomarkers, and clinical parameters (Table 3). However, the multidimensionality of these datasets presents substantial interpretive challenges. Machine-learning approaches applied to immune profiling and receptor-repertoire analysis have shown promise in disease stratification and outcome prediction [91,92]. Nevertheless, the lack of large, harmonized, longitudinal cohorts limits algorithm training and validation. In addition, regulatory considerations, data governance, and interpretability of predictive models must be addressed before large-scale clinical implementation. Establishing clinically validated frameworks for data integration remains an essential prerequisite for operationalizing endotype-driven precision strategies [93].

## 10. Disease-Specific B-Cell Signatures

**Figure 4:**
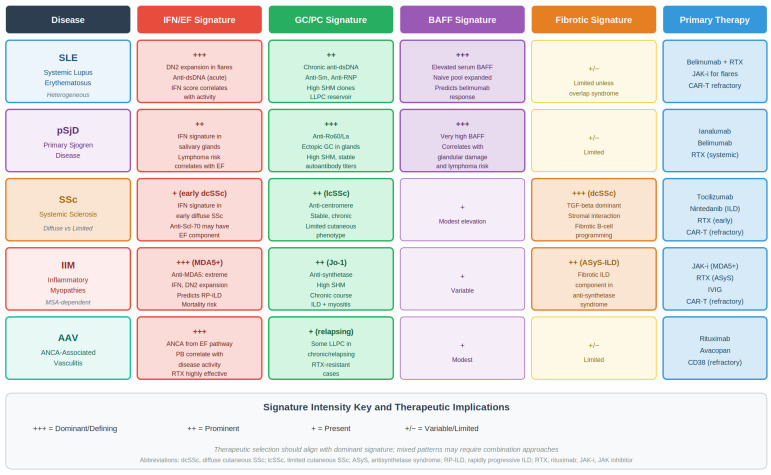
Disease-Specific B-Cell Endotype Signatures Across Systemic Autoimmune Diseases. Comparative overview of dominant B-cell-driven immunological architectures across major systemic autoimmune diseases. Intensity ratings reflect the relative predominance of four core immune programs: interferon-amplified extrafollicular responses (IFN/EF), germinal center and plasma cell-anchored activity (GC/PC), BAFF-dependent survival signaling, and fibro-inflammatory stromal programming. Systemic lupus erythematosus (SLE) displays marked endotype heterogeneity with co-dominance of IFN/EF and BAFF signatures. Primary Sjögren disease is characterized by prominent GC/PC and BAFF-dependent patterns associated with ectopic lymphoid structure formation. Systemic sclerosis segregates into fibro-dominant versus GC-anchored immune architectures according to autoantibody subtype. Inflammatory myopathies exhibit myositis-specific autoantibody-dependent immune programs, with anti-MDA5 disease showing extreme IFN-high/extrafollicular dominance. The figure emphasizes that therapeutic vulnerability reflects dominant immune circuitry rather than diagnostic category. Intensity scale: +++ dominant; ++ prominent; + present; +/− variable. This representation is a conceptual synthesis derived from human immunophenotyping datasets and is not intended as a deterministic classification. Abbreviations: AAV, ANCA-associated vasculitis; BAFF, B-cell activating factor; EF, extrafollicular; GC, germinal center; IFN, interferon; IIM, idiopathic inflammatory myopathies; MCTD, mixed connective tissue disease; MSA, myositis-specific autoantibodies; PC, plasma cell; pSjD, primary Sjögren disease; RP-ILD, rapidly progressive interstitial lung disease; SLE, systemic lupus erythematosus; SSc, systemic sclerosis; TLS, tertiary lymphoid structure.

Different autoimmune diseases exhibit characteristic patterns of B-cell endotype predominance, although substantial intra-disease heterogeneity persists (Figure 4) [1,89]. Understanding these disease-specific B-cell architectures helps explain divergent clinical trajectories and variable therapeutic responses.

In systemic lupus erythematosus, patients may cluster into interferon-high extrafollicular profiles with DN2 expansion and plasmablast surges during flares, while others show BAFF-dependent or germinal center/plasma cell-anchored profiles. These distinctions likely contribute to differential responses to B-cell depletion, BAFF inhibition, and interferon-targeted therapies [1,94,95].

Primary Sjögren disease is characterized by prominent germinal center/plasma cell signatures within salivary gland ectopic lymphoid structures, accompanied by elevated BAFF levels and microenvironments supporting plasma cell survival. These features align with the reported efficacy of BAFF-axis inhibitors for systemic autoantibody activity, whereas anti-CD20 therapy appears most effective for systemic manifestations rather than glandular dysfunction [96].

Systemic sclerosis displays subtype-specific B-cell profiles: diffuse cutaneous disease with anti-Scl-70 antibodies is often associated with stromal-conditioned, fibrosis-linked B-cell programs, while limited cutaneous disease with anti-centromere antibodies aligns more closely with germinal center/plasma cell-anchored signatures. This immunological divergence has therapeutic implications, particularly regarding the role of plasma cell-directed therapy versus antifibrotic strategies [97].

Inflammatory myopathies also demonstrate antibody-specific B-cell architectures. Anti-MDA5 dermatomyositis typically shows interferon-high extrafollicular activation consistent with responsiveness to JAK inhibition, while anti-synthetase syndrome exhibits more heterogeneous mixtures of germinal center, extrafollicular, and stromal-influenced programs [98,99,100].

Key differences between organ-specific and systemic autoimmune diseases include the degree of tissue-restricted versus multi-organ B-cell activation, the relative contribution of local versus systemic autoantibody production, and the extent of T-cell dependence [101,102]. Organ-specific diseases typically show stronger T-cell involvement in tissue destruction, whereas systemic diseases often demonstrate more prominent humoral dysregulation. However, the emerging recognition that both categories share common genetic susceptibility loci (CTLA4, PTPN22, IL2RA) and converge on similar B-cell activation pathways suggests that endotype-based frameworks may ultimately transcend the traditional organ-specific/systemic dichotomy, enabling mechanism-informed therapy selection across the full spectrum of autoimmune disease [102,103].

Myasthenia gravis provides a particularly instructive comparison, as it encompasses both IgG1/IgG3-mediated (acetylcholine receptor MG) and IgG4-mediated (MuSK MG) forms with fundamentally different pathomechanisms [104,105]. AChR-MG demonstrates complement-dependent damage at the neuromuscular junction, while MuSK-MG operates through direct receptor blockade without complement activation [75,105]. This dichotomy mirrors the IgG subclass-dependent heterogeneity discussed above and illustrates how the same clinical syndrome can arise from distinct immunological architectures requiring different therapeutic approaches.

In type 1 diabetes, B-cells contribute to disease through multiple mechanisms including autoantibody production against islet antigens (GAD65, IA-2, insulin, ZnT8) and antigen presentation to autoreactive T-cells [101,102]. The B-cell compartment in T1D shows features of both germinal center-derived and extrafollicular responses, with evidence of interferon signatures similar to those observed in SLE. Hashimoto’s thyroiditis demonstrates prominent intrathyroidal B-cell infiltration with formation of ectopic germinal centers, paralleling the tertiary lymphoid structures observed in salivary glands of Sjögren disease patients [103]. Both conditions show elevated CXCL13 expression, suggesting shared chemokine-driven B-cell recruitment mechanisms [101].

While this review focuses on systemic autoimmune diseases, comparison with organ-specific autoimmune conditions such as type 1 diabetes mellitus, Hashimoto’s thyroiditis, and myasthenia gravis reveals both shared mechanisms and distinctive features that inform the broader applicability of B-cell endotyping frameworks [101]. Organ-specific autoimmune diseases are characterized by immune attack restricted to a single tissue target, yet they share with systemic diseases the fundamental features of tolerance breakdown, autoreactive B- and T-cell activation, and autoantibody production.

Collectively, these observations support a model in which autoimmune diseases are not defined by fixed clinical categories but by reproducible immunological endotypes shaped by B-cell differentiation trajectories, tissue microenvironments, and cytokine pathways. Such endotype-based stratification provides a mechanistic foundation for precision immunotherapy.

## 11. Future Perspectives: Actionable Implementation Steps

Near-term (1–3 years): Plasmablast enumeration and basic B-cell subset profiling should be incorporated into the routine evaluation of patients with active systemic autoimmune disease using existing flow cytometry infrastructure. Serum BAFF measurement should be added to baseline assessment in SLE, primary Sjögren’s disease, and conditions where BAFF-targeted therapies are available. Interferon gene expression testing, already accessible through commercial platforms, should be considered in refractory patients to identify candidates for JAK inhibition or interferon-targeted therapy (Table 4). Finally, prospective sample collection and biobanking should be implemented to enable future integration of B-cell receptor sequencing as technologies mature.

Mid-term (3–5 years): Reference laboratory networks offering standardized B-cell endotyping panels should be established, supported by quality assurance programs and harmonized protocols. Clinical decision support tools integrating autoantibody profiles, cellular phenotypes, and serum biomarkers should be developed and validated. Prospective clinical trials comparing endotype-guided therapy with empirical treatment strategies in diseases such as SLE and inflammatory myopathies will be essential to demonstrate clinical utility. Routine integration of immune repertoire sequencing for refractory or relapsing disease should be introduced to track autoreactive clonotype evolution and therapeutic escape mechanisms.

Long-term (5–10 years): AI-driven endotype classification systems integrating clinical, serological, cellular, and molecular data should be implemented in clinical practice. Longitudinal immunomonitoring protocols enabling pre-emptive therapeutic intervention based on early pathway reactivation signals should become the standard of care. Regulatory approval of companion diagnostics guiding B-cell-targeted therapy selection will be critical for widespread adoption. Ultimately, combination therapeutic strategies integrating B-cell depletion with antigen-specific tolerization and immune reprogramming approaches may enable durable drug-free remission.

## 12. Conclusions

B-cells have undergone a conceptual renaissance over the past decade. Once viewed primarily as antibody-producing cells, they are now recognized as architects of immune microenvironments, regulators of chronic inflammation, and drivers of disease persistence through long-lived cellular and stromal interactions. Their differentiation trajectories—whether germinal center-derived, extrafollicular, BAFF-dependent, or TGF-β-conditioned—shape clinical expression, organ involvement, and therapeutic responsiveness across systemic autoimmune diseases.

The endotype framework proposed in this review—interferon-high/extrafollicular, germinal center/plasma cell-anchored, BAFF-driven/naïve-enriched, and TGF-β–fibrotic/stromal-interacting—provides a mechanistic bridge between B-cell biology and clinical decision-making. By mapping molecular circuitry to therapeutic vulnerability, it explains clinical heterogeneity that disease labels alone cannot capture and offers a biologically grounded rationale for treatment selection.

Implementation remains challenged by cost, standardization, data integration, and regulatory barriers, but these are increasingly addressable through the maturation of flow cytometry platforms, transcriptomic assays, and clinical bioinformatics. Complementary strategies, including T-cell modulation, FcRn inhibition, and antigen-specific tolerization, are likely to enhance outcomes when deployed alongside B-cell-directed therapies.

Over the next decade, systemic autoimmune diseases will likely be redefined not by syndromic labels but by dominant immune architectures. Understanding B-cell biology is no longer optional. It is the foundation for precision immunotherapy, durable remission, and a new clinical paradigm in which treatment is guided by immune circuitry rather than empiric escalation.

## Figures and Tables

**Table 1:** Autoantibody–B-Cell Endotype Mapping.

Autoantibody	Dominant B-Cell Endotype	Typical Clinical Associations	Key Immunobiological Features	Therapeutic Vulnerabilities (Context-Dependent)
Anti-MDA5	IFN-high/Extrafollicular (EF-biased)	Dermatomyositis, RP-ILD	Low SHM, DN2/ABC expansion, high IFN signature, plasmablast bursts	JAK inhibitors, glucocorticoids, RTX (case-series and emerging evidence)
Anti-U1RNP	IFN-high/EF-enriched	MCTD, overlap syndromes, vasculopathy	IFN-driven activation, plasmablast bias, Raynaud association	IFN-pathway targeting, JAK inhibitors, immunosuppression
Anti-Ro52 (TRIM21)	IFN-high/EF-enriched	pSjD, IIM, SSc-ILD	Type I/II IFN activation, vascular tropism, lung involvement	High-intensity immunosuppression in selected severe phenotypes
Anti-centromere	Germinal Center/Plasma Cell-anchored	Limited SSc, PAH	High SHM, LLPC persistence, BM niching	Supportive care; B-cell targeting under investigation
Anti-Ro60/Anti-La (SS-A/B)	GC/LLPC-dominant	pSjD, SLE, NLE	LLPC niches, structured TLS, chronic autoantibody production	BAFF-R targeting (investigational), plasma-cell modulation (experimental)
Anti-Scl-70 (Topo-I)	Tissue-conditioned (fibro-inflammatory)	Diffuse SSc, ILD	Stromal imprinting, TLS presence, ECM remodeling	Antifibrotics, IL-6 blockade; B-cell targeting under investigation
RF/Anti-CCP	Mixed GC/BAFF-modulated	Seropositive RA	GC activity, BAFF sensitivity, transitional B-cell expansion	B-cell depletion, costimulation blockade; BAFF inhibition in subsets

This table summarizes the association between major autoantibody specificities and dominant B-cell endotypes inferred from human immunophenotyping, single-cell analyses, immune repertoire profiling and tissue studies. Endotype assignments reflect statistically enriched immunobiological contexts rather than deterministic lineage attribution. Therapeutic vulnerabilities indicate mechanistic susceptibilities rather than treatment recommendations. Abbreviations: ABC, age-associated B-cell; BAFF, B-cell activating factor; GC, germinal center; EF, extrafollicular; BM, bone marrow; DN2, double-negative 2 B-cell; ECM, extracellular matrix; IFN, interferon; IIM, inflammatory idiopathic myopathy; ILD, interstitial lung disease; LLPC, long-lived plasma cell; MCTD, mixed connective tissue disease; NLE, neonatal lupus erythematosus; PAH, pulmonary arterial hypertension; pSjD, primary Sjögren disease; RA, rheumatoid arthritis; RP-ILD, rapidly progressive interstitial lung disease; RTX, rituximab; SLE, systemic lupus erythematosus; SSc, systemic sclerosis; SHM, somatic hypermutation; TLS, tertiary lymphoid structure.

**Table 2:** Endotype-Mechanism Alignment of B-Cell-Directed Therapeutic Strategies.

Biological Target	Mechanism	Endotype Association	Biological Limitation
Mature B-cells (CD20)	Depletion of antigen-experienced B-cells	Memory-dominant/GC-associated endotypes	Spares plasma cells; rapid serological rebound
BAFF survival axis	Disruption of transitional and naïve B-cell survival	BAFF-high survival-biased architectures	Slow pharmacodynamic effect
IFN signaling hubs (JAK–STAT)	Inhibition of inflammatory license	IFN-high/extrafollicular endotypes	Indirect effect on B-cells
Plasma cells (CD38/proteasome)	Disruption of antibody secretion machinery	Plasma-cell-anchored architectures	Niche protection limits durability
B-cell lineage (CD19)	Immune circuit collapse	Refractory immune configurations	Immune toxicity risk

Rather than listing individual drugs, this table summarizes biologically meaningful therapeutic classes according to their dominant cellular target, associated immune architecture, and intrinsic mechanistic limitations. The framework emphasizes biological vulnerability rather than clinical indication. Abbreviations: BAFF, B-cell activating factor; GC, germinal center; IFN, interferon; JAK, Janus kinase.

**Table 3:** Clinical Biomarkers for B-Cell Endotype Assessment.

Biomarker	Definition/Panel	Endotype Indication	Clinical Utility	Availability
DN2 B-cells	CD19^+^CD27^−^IgD^−^CD11c^+^ T-bet^+^	IFN-high/EF	Flare risk; immune pathway stratification	Specialized flow cytometry
Plasmablasts	CD19^+^CD27^++^CD38^++^ CD20^−^/low	Active immune circuit	Disease activity monitoring	Standard flow cytometry
Type I IFN score	ISG expression profile (e.g., IFI27, IFI44, IFIT1, ISG15)	IFN-high endotype	IFN-pathway activity	Commercial immune panels
Serum BAFF	ELISA-based quantification	BAFF-driven endotype	BAFF-axis activation	Reference laboratories
Free light chains	κ, λ, κ/λ ratio	Plasma-cell-anchored	Plasma-cell activity marker	Routine laboratories

Abbreviations: EF, extrafollicular; IFN, interferon; ISG, interferon-stimulated gene; BAFF, B-cell activating factor.

**Table 4:** B-Cell Endotype–Therapy Mapping for Precision Treatment Selection.

Endotype	Key Biological Identifiers	First-Line Therapies	Refractory Options	Avoid/Limited Benefit
IFN-high/EF	DN2 expansion, T-bet^+^ plasmablasts, high IFN score	JAK inhibitors + rituximab	CAR-T, anti-CD19, anifrolumab	Belimumab alone; plasma cell-directed therapy
GC/Plasma cell-anchored	High SHM autoantibodies, stable titers	Daratumumab, bortezomib	CAR-T, BCMA-targeting	Rituximab alone
BAFF-driven	High BAFF, transitional and naïve B-cell expansion	Belimumab + rituximab	Ianalumab, anti-CD19	Rituximab monotherapy
TGF-β-fibrotic	Anti-Scl-70, fibrosis biomarkers	Nintedanib, tocilizumab	Anti-CD19, CAR-T, HSCT	B-cell depletion alone

Abbreviations: BAFF, B-cell activating factor; EF, extrafollicular; GC, germinal center; HSCT, hematopoietic stem cell transplantation; IFN, interferon; SHM, somatic hypermutation.

## Data Availability

No new data were created or analyzed in this study.

## Abbreviations

ABC	Age-associated B-cell
AAV	ANCA-associated vasculitis
AI	Artificial intelligence
APRIL	A proliferation-inducing ligand
BAFF	B-cell activating factor
BAFF-R	BAFF receptor
BCMA	B-cell maturation antigen
BCR	B-cell receptor
BM	Bone marrow
CAR-T	Chimeric antigen receptor T-cell
CCL	Chemokine (C–C motif) ligand
CD	Cluster of differentiation
CDC	Complement-dependent cytotoxicity
CRS	Cytokine release syndrome
DN2	Double-negative 2 B-cell
DsDNA	Double-stranded DNA
ECM	Extracellular matrix
EF	Extrafollicular
ELISA	Enzyme-linked immunosorbent assay
FcRn	Neonatal Fc receptor
FDC	Follicular dendritic cell
GC	Germinal center
GOF	Gain of function
HSCT	Hematopoietic stem cell transplantation
IIM	Idiopathic inflammatory myopathy
IFN	Interferon
ISG	Interferon-stimulated gene
JAK	Janus kinase
LLPC	Long-lived plasma cell
MCTD	Mixed connective tissue disease
MHC	Major histocompatibility complex
MSA	Myositis-specific autoantibodies
mTOR	Mechanistic target of rapamycin
NLE	Neonatal lupus erythematosus
NK	Natural killer cell
PAH	Pulmonary arterial hypertension
PB	Plasmablast
PC	Plasma cell
pSjD	Primary Sjögren’s disease
RA	Rheumatoid arthritis
RNA-seq	RNA sequencing
RP-ILD	Rapidly progressive interstitial lung disease
RTX	Rituximab
ScRNA-seq	Single-cell RNA sequencing
SDC	Syndecan 1 (CD138)
SHM	Somatic hypermutation
SLE	Systemic lupus erythematosus
SSc	Systemic sclerosis
STAT	Signal transducer and activator of transcription
TCR	T-cell receptor
Tfh	T follicular helper cell
TGF-β	Transforming growth factor beta
TLS	Tertiary lymphoid structure
TLR	Toll-like receptor
Treg	Regulatory T-cell
V(D)J	Variable, diversity and joining
VTE	Venous thromboembolism

## References

[1] Shlomchik M.J. (2008). Sites and stages of autoreactive B cell activation and regulation. Immunity.

[2] Rawlings D.J., Metzler G., Wray-Dutra M., Jackson S.W. (2017). Altered B cell signalling in autoimmunity. Nat. Rev. Immunol..

[3] Dorner T., Jacobi A.M., Lipsky P.E. (2009). B cells in autoimmunity. Arthritis Res. Ther..

[4] Corsiero E., Nerviani A., Bombardieri M., Pitzalis C. (2016). Ectopic Lymphoid Structures: Powerhouse of Autoimmunity. Front. Immunol..

[5] Asam S., Nayar S., Gardner D., Barone F. (2021). Stromal cells in tertiary lymphoid structures: Architects of autoimmunity. Immunol. Rev..

[6] Cyster J.G., Allen C.D.C. (2019). B Cell Responses: Cell Interaction Dynamics and Decisions. Cell.

[7] Elsner R.A., Shlomchik M.J. (2020). Germinal Center and Extrafollicular B Cell Responses in Vaccination, Immunity, and Autoimmunity. Immunity.

[8] Jenks S.A., Cashman K.S., Zumaquero E., Marigorta U.M., Patel A.V., Wang X., Tomar D., Woodruff M.C., Simon Z., Bugrovsky R. (2018). Distinct Effector B Cells Induced by Unregulated Toll-like Receptor 7 Contribute to Pathogenic Responses in Systemic Lupus Erythematosus. Immunity.

[9] Victora G.D., Nussenzweig M.C. (2012). Germinal centers. Annu. Rev. Immunol..

[10] Mesin L., Ersching J., Victora G.D. (2016). Germinal Center B Cell Dynamics. Immunity.

[11] Bannard O., Cyster J.G. (2017). Germinal centers: Programmed for affinity maturation and antibody diversification. Curr. Opin. Immunol..

[12] Eisenbarth S.C., Batista F., Cyster J., Elsner R., Kelsoe G., Lund F.E., Shlomchik M.J., Sweet R.A., Vinuesa C.G., Bhattacharya D. (2025). A roadmap for defining “extrafollicular” B cell responses. Immunity.

[13] Pipi E., Nayar S., Gardner D.H., Colafrancesco S., Smith C., Barone F. (2018). Tertiary Lymphoid Structures: Autoimmunity Goes Local. Front. Immunol..

[14] Rivellese F., Mauro D., Nerviani A., Pagani S., Fossati-Jimack L., Messemaker T.C., Clark E., Rosber M.K., Lewis M., Bornstein S. (2018). Mast cells in early rheumatoid arthritis associate with disease severity and support B cell autoantibody production. Ann. Rheum. Dis..

[15] Mueller S.N., Germain R.N. (2009). Stromal cell contributions to the homeostasis and functionality of the immune system. Nat. Rev. Immunol..

[16] Arvidsson G., Czarnewski P., Johansson A., Raine A., Imgenberg-Kreuz J., Nordlund J., Petri A., Svenungsson E., Cavalli G., Nordmark G. (2024). Multimodal Single-Cell Sequencing of B Cells in Primary Sjogren’s Syndrome. Arthritis Rheumatol..

[17] Su C., Wang W., Cheng F., Zhao F., Zheng S.G. (2025). The role of B cells in Sjogren’s syndrome and their impact on the nervous system. Autoimmun. Rev..

[18] Wu C., Jiang S., Chen Z., Li T., Gu X., Dai M., Chen X., Zhang L., Tang H., Zhou J. (2024). Single-cell transcriptomics reveal potent extrafollicular B cell response linked with granzyme K(+) CD8 T cell activation in lupus kidney. Ann. Rheum. Dis..

[19] Zhao J., Bai X., Zhou C., Ouyang Q., Zhang Y., Zhang X., Wang H., Li Z., Chen M., Liu Y. (2025). Cxcl9(high) macrophages recruit circulating Cxcr3+ plasmablasts into kidneys to promote pathogenesis of lupus nephritis mice. Commun. Biol..

[20] Jenks S.A., Cashman K.S., Woodruff M.C., Lee F.E., Sanz I. (2019). Extrafollicular responses in humans and SLE. Immunol. Rev..

[21] Brown G.J., Canete P.F., Wang H., Medhavy A., Bones J., Roco J.A., He Y., Lawber K.E., Burgio G., Almeida-Santos J. (2022). TLR7 gain-of-function genetic variation causes human lupus. Nature.

[22] Mackay F., Figgett W.A., Saulep D., Lepage M., Hibbs M.L. (2010). B-cell stage and context-dependent requirements for survival signals from BAFF and the B-cell receptor. Immunol. Rev..

[23] Meffre E., O’Connor K.C. (2019). Impaired B-cell tolerance checkpoints promote the development of autoimmune diseases and pathogenic autoantibodies. Immunol. Rev..

[24] Li J., Zhao M., Luo W., Huang J., Zhao B., Zhou Z. (2023). B cell metabolism in autoimmune diseases: Signaling pathways and interventions. Front. Immunol..

[25] Boothby M., Rickert R.C. (2017). Metabolic Regulation of the Immune Humoral Response. Immunity.

[26] Victora G.D., Nussenzweig M.C. (2022). Germinal Centers. Annu. Rev. Immunol..

[27] Radbruch A., Muehlinghaus G., Luger E.O., Inamine A., Smith K.G., Dorner T., Heer A.K., Berek C. (2006). Competence and competition: The challenge of becoming a long-lived plasma cell. Nat. Rev. Immunol..

[28] Moser K., Tokoyoda K., Radbruch A., MacLennan I., Manz R.A. (2006). Stromal niches, plasma cell differentiation and survival. Curr. Opin. Immunol..

[29] Pyzik M., Kozicky L.K., Gandhi A.K., Blumberg R.S. (2023). The therapeutic age of the neonatal Fc receptor. Nat. Rev. Immunol..

[30] Xiang N., Xu H., Zhou Z., Wang J., Cai P., Wang L., Tang J., Wang X., Liu C., Chen S. (2023). Single-cell transcriptome profiling reveals immune and stromal cell heterogeneity in primary Sjogren’s syndrome. iScience.

[31] Huang J., Tang J., Zhang C., Liu T., Deng Z., Liu L. (2024). Single-cell transcriptomic analysis uncovers heterogeneity in the labial gland microenvironment of primary Sjogren’s syndrome. J. Transl. Autoimmun..

[32] Inamo J., Takeshita M., Suzuki K., Tsunoda K., Usuda S., Kuramoto J., Moody J., Hon C.-C., Ando Y., Sasaki T. (2025). Comparative single-cell and spatial profiling of anti-SSA-positive and anti-centromere-positive Sjogren’s disease reveals common and distinct immune activation and fibroblast-mediated inflammation. Nat. Commun..

[33] Pitzalis C., Jones G.W., Bombardieri M., Jones S.A. (2014). Ectopic lymphoid-like structures in infection, cancer and autoimmunity. Nat. Rev. Immunol..

[34] Dong Y., Wang T., Wu H. (2023). Tertiary lymphoid structures in autoimmune diseases. Front. Immunol..

[35] Guillaume S.M., Beccaria C.G., Iannacone M., Linterman M.A. (2025). Tertiary Lymphoid Structures Across Organs: Context, Composition, and Clinical Levers. Immunol. Rev..

[36] Zou M., Qian D., Luo R., Cheng Y., Xu G., Ge S. (2025). Identifying potential mechanism and targets for treatment of tertiary lymphoid structure in lupus nephritis based on bioinformatics analysis. Int. Immunopharmacol..

[37] Wang Q., Feng D., Jia S., Lu Q., Zhao M. (2024). B-Cell Receptor Repertoire: Recent Advances in Autoimmune Diseases. Clin. Rev. Allergy Immunol..

[38] Deguine J., Xavier R.J. (2024). B cell tolerance and autoimmunity: Lessons from repertoires. J. Exp. Med..

[39] Ota M., Nakano M., Nagafuchi Y., Kobayashi S., Hatano H., Yoshida R., Akutsu Y., Itamiya T., Ban N., Tsuchida Y. (2023). Multimodal repertoire analysis unveils B cell biology in immune-mediated diseases. Ann. Rheum. Dis..

[40] Wilbrink R., van der Weele L., Spoorenberg A.J.P.L., de Vries N., Niewold I.T.G., Verstappen G.M., Kroese F.G.M. (2025). B Cell Receptor Repertoire Analysis of the CD21(lo) B Cell Compartment in Healthy Individuals, Patients With Sjogren’s Disease, and Patients With Radiographic Axial Spondyloarthritis. Eur. J. Immunol..

[41] Zaslavsky M.E., Craig E., Michuda J.K., Sehgal N., Ram-Mohan N., Lee J.Y., Nguyen K.D., Hoh R.A., Pham T.D., Röltgen K. (2025). Disease diagnostics using machine learning of B cell and T cell receptor sequences. Science.

[42] Voss L.F., Howarth A.J., Wittenborn T.R., Hummelgaard S., Juul-Madsen K., Kastberg K.S., Pedersen M.K., Jensen L., Papanastasiou A.D., Vorup-Jensen T. (2022). The extrafollicular response is sufficient to drive initiation of autoimmunity and early disease hallmarks of lupus. Front. Immunol..

[43] Al-Aubodah T.A., Aoudjit L., Pascale G., Perinpanayagam M.A., Langlais D., Bhargava R., Bhalla N., Lee S.J., Liu Q., Bhalla A. (2023). The extrafollicular B cell response is a hallmark of childhood idiopathic nephrotic syndrome. Nat. Commun..

[44] Zhu D.Y., Maurer D.P., Castrillon C., Deng Y., Mohamed F.A.N., Ma M., Schuck P., Bhargava R., Li X., He L. (2025). CD21 primes extrafollicular differentiation of autoreactive B cells in a TLR7-driven lupus model. Sci. Immunol..

[45] Horisberger A., Griffith A., Keegan J., Arazi A., Pulford J., Murzin E., Cordoba S., Hacohen N., Diamond B., Bhalla A. (2025). Blood immunophenotyping identifies distinct kidney histopathology and outcomes in patients with lupus nephritis. J. Clin. Investig..

[46] Wan L., Guo J., Sun A., Chen H., Hu B., Liu C. (2025). The application value of peripheral plasmablasts in the assessment of disease activity and treatment response in systemic lupus erythematosus. Clin. Exp. Rheumatol..

[47] Jacobi A.M., Odendahl M., Reiter K., Bruns A., Burmester G.R., Radbruch A., Voll R.E., Dorner T. (2003). Correlation between circulating CD27high plasma cells and disease activity in patients with systemic lupus erythematosus. Arthritis Rheum..

[48] Peng Y., Guo F., Liao S., Liao H., Xiao H., Yang L., Li X., Wang X., Zhang J., Chen L. (2020). Altered frequency of peripheral B-cell subsets and their correlation with disease activity in patients with systemic lupus erythematosus: A comprehensive analysis. J. Cell. Mol. Med..

[49] Xie G., Chen X., Gao Y., Yang M., Zhou S., Lu L., Tian R., Wang X., Li Y., Zhang Z. (2025). Age-Associated B Cells in Autoimmune Diseases: Pathogenesis and Clinical Implications. Clin. Rev. Allergy Immunol..

[50] Huang W., Quach T.D., Dascalu C., Liu Z., Leung T., Byrne-Steele M., Bhargava A., Bhalla R., Bhalla A., Han S. (2018). Belimumab promotes negative selection of activated autoreactive B cells in systemic lupus erythematosus patients. JCI Insight.

[51] Malkiel S., Barlev A.N., Atisha-Fregoso Y., Suurmond J., Diamond B. (2018). Plasma Cell Differentiation Pathways in Systemic Lupus Erythematosus. Front. Immunol..

[52] Suurmond J., Diamond B. (2015). Autoantibodies in systemic autoimmune diseases: Specificity and pathogenicity. J. Clin. Investig..

[53] Hiepe F., Radbruch A. (2016). Plasma cells as an innovative target in autoimmune disease with renal manifestations. Nat. Rev. Nephrol..

[54] Dorner T., Lipsky P.E. (2016). Beyond pan-B-cell-directed therapy—New avenues and insights into the pathogenesis of SLE. Nat. Rev. Rheumatol..

[55] Edwards J.C., Szczepanski L., Szechinski J., Filipowicz-Sosnowska A., Emery P., Close D.R., Stevens R.M., Shaw T. (2004). Efficacy of B-cell-targeted therapy with rituximab in patients with rheumatoid arthritis. N. Engl. J. Med..

[56] Hauser S.L., Bar-Or A., Comi G., Giovannoni G., Hartung H.P., Hemmer B., Lublin F., Montalban X., Rammohan K.W., Selmaj K. (2017). Ocrelizumab versus Interferon Beta-1a in Relapsing Multiple Sclerosis. N. Engl. J. Med..

[57] Hiepe F., Dorner T., Hauser A.E., Hoyer B.F., Mei H., Radbruch A. (2011). Long-lived autoreactive plasma cells drive persistent autoimmune inflammation. Nat. Rev. Rheumatol..

[58] Mackay F., Schneider P. (2009). Cracking the BAFF code. Nat. Rev. Immunol..

[59] Mackay F., Browning J.L. (2002). BAFF: A fundamental survival factor for B cells. Nat. Rev. Immunol..

[60] Navarra S.V., Guzman R.M., Gallacher A.E., Hall S., Levy R.A., Jimenez R.E., Li E.K., Thomas M., Kim H.Y., Leon M.G. (2011). Efficacy and safety of belimumab in patients with active systemic lupus erythematosus: A randomised, placebo-controlled, phase 3 trial. Lancet.

[61] Dorner T., Kinnman N., Tak P.P. (2010). Targeting B cells in immune-mediated inflammatory disease: A comprehensive review of mechanisms of action and identification of biomarkers. Pharmacol. Ther..

[62] O’Shea J.J., Plenge R. (2012). JAK and STAT signaling molecules in immunoregulation and immune-mediated disease. Immunity.

[63] Zhu S., Li S., Shi L., Chu T., Huang Z., Lu X., Wang Y., Zhang L., Chen H., Li M. (2025). Baricitinib could improve the prognosis of anti-MDA5 antibody positive dermatomyositis associated interstitial lung disease. Arthritis Res. Ther..

[64] Yanagihara T., Mirza R.D., Kolb M.R.J. (2025). Tofacitinib in anti-MDA5-positive dermatomyositis-associated interstitial lung disease: A new standard of care emerges. Eur. Respir. J..

[65] Alexander T., Sarfert R., Klotsche J., Kuhl A.A., Rubbert-Roth A., Lorenz H.M., Rech J., Schleenvoigt B.T., Arnold R., Blaschek B. (2015). The proteasome inhibitior bortezomib depletes plasma cells and ameliorates clinical manifestations of refractory systemic lupus erythematosus. Ann. Rheum. Dis..

[66] Manz R.A., Hauser A.E., Hiepe F., Radbruch A. (2005). Maintenance of serum antibody levels. Annu. Rev. Immunol..

[67] Schroeder H.W., Cavacini L. (2010). Structure and function of immunoglobulins. J. Allergy Clin. Immunol..

[68] Mackensen A., Muller F., Mougiakakos D., Boltz S., Wilhelm A., Aigner M., Völkl S., Simon D., Kleyer A., Munoz L. (2022). Anti-CD19 CAR T cell therapy for refractory systemic lupus erythematosus. Nat. Med..

[69] Muller F., Taubmann J., Bucci L., Wilhelm A., Bergmann C., Volkl S., Aigner M., Rothe T., Minopoulou I., Tur C. (2024). CD19 CAR T-Cell Therapy in Autoimmune Disease—A Case Series with Follow-up. N. Engl. J. Med..

[70] Chung J.B., Brudno J.N., Borie D., Kochenderfer J.N. (2024). Chimeric antigen receptor T cell therapy for autoimmune disease. Nat. Rev. Immunol..

[71] Kansal R., Richardson N., Neeli I., Khawaja S., Chamberlain D., Ghani M., Ghazi M., Soto H., Bhargava A., Bhalla A. (2019). Sustained B cell depletion by CD19-targeted CAR T cells is a highly effective treatment for murine lupus. Sci. Transl. Med..

[72] Vidarsson G., Dekkers G., Rispens T. (2014). IgG subclasses and allotypes: From structure to effector functions. Front. Immunol..

[73] Rispens T., Huijbers M.G. (2023). The unique properties of IgG4 and its roles in health and disease. Nat. Rev. Immunol..

[74] Khosroshahi A., Carruthers M.N., Deshpande V., Unizony S., Bloch D.B., Stone J.H. (2012). Rituximab for the treatment of IgG4-related disease: Lessons from 10 consecutive patients. Medicine.

[75] Huijbers M.G., Plomp J.J., van der Maarel S.M., Verschuuren J.J. (2018). IgG4-mediated autoimmune diseases: A niche of antibody-mediated disorders. Ann. N. Y. Acad. Sci..

[76] Zhang H., Li P., Wu D., Xu D., Hou Y., Wang Q., Li M., Li Y., Zeng X., Zhang F. (2015). Serum IgG subclasses in autoimmune diseases. Medicine.

[77] Crotty S. (2019). T Follicular Helper Cell Biology: A Decade of Discovery and Diseases. Immunity.

[78] Sakaguchi S., Yamaguchi T., Nomura T., Ono M. (2008). Regulatory T cells and immune tolerance. Cell.

[79] Rosenzwajg M., Churlaud G., Mallone R., Six A., Derian N., Chaara W., Lorenzon R., Long S.A., Buckner J.H., Afonso G. (2015). Low-dose interleukin-2 fosters a dose-dependent regulatory T cell tuned milieu in T1D patients. J. Autoimmun..

[80] Roopenian D.C., Akilesh S. (2007). FcRn: The neonatal Fc receptor comes of age. Nat. Rev. Immunol..

[81] Zhu L.N., Hou H.M., Wang S., Zhang S., Wang G.G., Guo Z.Y., Wang X., Li Y., Chen Z., Zhang J. (2023). FcRn inhibitors: A novel option for the treatment of myasthenia gravis. Neural Regen. Res..

[82] LaMothe R.A., Kolte P.N., Vo T., Ferrari J.D., Gelsinger T.C., Wong J., Chan V.T., Ahmed S., Srinivasan A., Deitemeyer P. (2018). Tolerogenic Nanoparticles Induce Antigen-Specific Regulatory T Cells and Provide Therapeutic Efficacy and Transferrable Tolerance against Experimental Autoimmune Encephalomyelitis. Front. Immunol..

[83] Bluestone J.A., Buckner J.H., Herold K.C. (2021). Immunotherapy: Building a bridge to a cure for type 1 diabetes. Science.

[84] Kenison J.E., Jhaveri A., Li Z., Khadse N., Tjon E., Tezza S., Mayo L., Bhalla A., Bhargava R., Quintana F.J. (2020). Tolerogenic nanoparticles suppress central nervous system inflammation. Proc. Natl. Acad. Sci. USA.

[85] Hasin Y., Seldin M., Lusis A. (2017). Multi-omics approaches to disease. Genome Biol..

[86] Stark R., Grzelak M., Hadfield J. (2019). RNA sequencing: The teenage years. Nat. Rev. Genet..

[87] See P., Lum J., Chen J., Ginhoux F. (2018). A Single-Cell Sequencing Guide for Immunologists. Front. Immunol..

[88] van Dongen J.J.M., Lhermitte L., Bottcher S., Almeida J., van der Velden V.H.J., Flores-Montero J., Rawstron A., Asnafi V., Lecrevisse Q., Lucio P. (2012). EuroFlow antibody panels for standardized n-dimensional flow cytometric immunophenotyping of normal, reactive and malignant leukocytes. Leukemia.

[89] Solly F., Angelot-Delettre F., Ticchioni M., Genevieve F., Rambaud H., Baseggio L., Plesa A., Debliquis A., Garnache-Ottou F., Roggy A. (2019). Standardization of Flow Cytometric Immunophenotyping for Hematological Malignancies: The FranceFlow Group Experience. Cytom. Part A.

[90] Kelleher P., Greathead L., Whitby L., Brando B., Barnett D., Bloxham D., Detute R., Dunlop A., Farren T., UK NEQAS Leucocyte Immunophenotyping Steering Committee (2024). European flow cytometry quality assurance guidelines for the diagnosis of primary immune deficiencies and assessment of immune reconstitution following B cell depletion therapies and transplantation. Cytom. Part B Clin. Cytom..

[91] Greiff V., Menzel U., Haessler U., Cook S.C., Friedensohn S., Khan T.A., Pogson M., Hellmann I., Reddy S.T. (2014). Quantitative assessment of the robustness of next-generation sequencing of antibody variable gene repertoires from immunized mice. BMC Immunol..

[92] Shemesh O., Polak P., Lundin K.E.A., Sollid L.M., Yaari G. (2021). Machine Learning Analysis of Naive B-Cell Receptor Repertoires Stratifies Celiac Disease Patients and Controls. Front. Immunol..

[93] Esteva A., Robicquet A., Ramsundar B., Kuleshov V., DePristo M., Chou K., Cui C., Corrado G., Thrun S., Dean J. (2019). A guide to deep learning in healthcare. Nat. Med..

[94] Dorner T., Lipsky P.E. (2014). B cells: Depletion or functional modulation in rheumatic diseases. Curr. Opin. Rheumatol..

[95] Fillatreau S., Manfroi B., Dorner T. (2021). Toll-like receptor signalling in B cells during systemic lupus erythematosus. Nat. Rev. Rheumatol..

[96] Bombardieri M., Lewis M., Pitzalis C. (2017). Ectopic lymphoid neogenesis in rheumatic autoimmune diseases. Nat. Rev. Rheumatol..

[97] Thoreau B., Chaigne B., Mouthon L. (2022). Role of B-Cell in the Pathogenesis of Systemic Sclerosis. Front. Immunol..

[98] Reyes-Huerta R.F., Mandujano-Lopez V., Velasquez-Ortiz M.G., Alcala-Carmona B., Ostos-Prado M.J., Reyna-Juarez Y., Garcia-Blanco A., Maldonado-Bernal C., Meza-Sanchez D., Llorente-Garcia L. (2024). Novel B-cell subsets as potential biomarkers in idiopathic inflammatory myopathies: Insights into disease pathogenesis and disease activity. J. Leukoc. Biol..

[99] Franco C., Gatto M., Iaccarino L., Ghirardello A., Doria A. (2021). Lymphocyte immunophenotyping in inflammatory myositis: A review. Curr. Opin. Rheumatol..

[100] Pan Z., Li M., Zhang P., Li T., Liu R., Liu J., Wang Y., Chen H., Zhang L., Li X. (2025). Peripheral Blood Lymphocyte Subsets and Heterogeneity of B Cell Subsets in Patients of Idiopathic Inflammatory Myositis with Different Myositis-specific Autoantibodies. Inflammation.

[101] Li L., Liu S., Yu J. (2020). Autoimmune thyroid disease and type 1 diabetes mellitus: Same pathogenesis; new perspective?. Ther. Adv. Endocrinol. Metab..

[102] Naser S.S., Mahdi B.M. (2021). Type 1 diabetes and autoimmune thyroid disease—The genetic link. Front. Endocrinol..

[103] Ragusa F., Fallahi P., Elia G., Gonnella D., Paparo S.R., Giusti C., Churilov L.P., Ferrari S.M., Antonelli A. (2019). Hashimoto thyroiditis: Epidemiology, pathogenesis, clinic and therapy. Best Pract. Res. Clin. Endocrinol. Metab..

[104] Gilhus N.E., Verschuuren J.J. (2015). Myasthenia gravis: Subgroup classification and therapeutic strategies. Lancet Neurol..

[105] Koneczny I., Herbst R. (2019). Myasthenia gravis: Pathogenic effects of autoantibodies on neuromuscular architecture. Cells.

